# Concerns regarding interpretation and reporting in Kolivas et al. mHealth low carbohydrate dietary intervention in T2D

**DOI:** 10.1038/s44324-026-00105-5

**Published:** 2026-04-24

**Authors:** J. Dening

**Affiliations:** Unaffiliated, Brisbane, QLD Australia

**Keywords:** Diseases, Endocrinology, Health care, Medical research

Kolivas et al. recently reported the outcomes of a three-month mHealth low carbohydrate dietary intervention for people with type 2 diabetes (T2D)^1^. Given the limited evidence for digital dietary interventions for T2D, particularly in the Australian context, this is a commendable contribution to an important field. However, several aspects of data interpretation and reporting require clarification or correction.

At three months, this single-arm pre-post intervention demonstrated significant reductions in haemoglobin A1c (HbA1c) (−1.0%), fasting plasma glucose (FPG) (−1.3 mmol/L) and systolic blood pressure (−6 mmHg), with no significant changes in diastolic blood pressure, body weight, body mass index (BMI) or waist circumference. In contextualising the findings, the authors cite the T2Diet Study^2^, the only randomised controlled trial of a digital low carbohydrate dietary program for T2D conducted in Australia. They accurately reported the between-group differences of the 16-week intervention—HbA1c −0.6%, body weight −3.3 kg, BMI −1.1 kg/m², and reduced medication requirements—but then misrepresent these results as within-group changes, stating that “while the average reduction in HbA1c in the T2Diet study (−0.6%) was less than the improvement recorded in the current trial (−1.0%)…” (p. 6). This comparison understates the T2Diet findings and exaggerates the effect of the current intervention. In fact, the T2Diet within-group changes were HbA1c −0.94%, weight −4.36 kg, and BMI −1.48 kg/m²—all statistically significant. Distinguishing between-group differences from within-group changes is essential for fair comparison and accurate scientific and clinical interpretation^3,4^. Being a single-arm trial reporting within-group changes, the manuscript should clearly reflect this distinction to prevent misrepresentation of relative intervention effects.

In several parts of the manuscript, the authors present statistically non-significant findings as significant, creating potential for misinterpretation. For example, HbA1c changes between predefined carbohydrate intake groups (≤50 g versus >50 g/day) were assessed. The results on adherence (p. 4) reported a non-significant HbA1c reduction of −0.5% (95% CI: −1.0 to 0.0; p = 0.073; Supplementary Table S3)^1^, suggesting no association between the level of carbohydrate intake and HbA1c reduction in the cohort. However, the report then contradicts this finding by stating that “participants who reduced their HbA1c…significantly reduced their dietary carbohydrates when grouped by intake of 50 g a day or less…(*p* = 0.036)”—with no additional supporting data. Likewise, multiple statements in the abstract (p. 1), discussion (p. 5), limitations and conclusion (p. 8), described this outcome as a significant finding, implying that participants who consumed a lower carbohydrate intake had greater reductions in HbA1c—claims that do not appear to be supported by the reported analysis. Such discrepancies contravene recommended reporting practices, which emphasise that non-significant results should be described transparently and not implied to show benefit^3–5^.

Similarly, weight outcomes are misinterpreted. In the trial’s secondary outcome analysis for anthropometric markers—BMI, waist circumference, and body weight did not change significantly in the full sample (n = 99; p = 0.200). Nonetheless, the authors conducted a post-hoc subgroup analysis with 92 participants stratified by ≥5% versus <5% weight loss (Supplementary Table S2)^1^. Conducting a subgroup analysis when the overall result is non-significant, especially with unexplained exclusion of 7 participants, is unusual unless such analyses were pre-specified, which does not appear to be outlined in the study methods^1^ or trial protocol^6^. While the authors reported the majority of participants numerically lost weight (89%), the overall intervention effect was non-significant. It is recognised that a modest weight loss of 5–10% can provide clinical benefits in the management of T2D^7^. However, only around one third of participants achieved ≥5% weight loss. Therefore, emphasising these subgroup outcomes in the conclusion as a “clinically significant reduction in body weight” (p. 8) is misleading and risks overstating intervention efficacy to clinicians and patients. Recommended reporting practices caution that the use of post-hoc subgroup analyses should be clearly distinguished from primary outcomes and interpreted with caution to avoid giving a false impression of treatment effect^3–5^.

In summary, the issues identified—misrepresentation of comparative data, misinterpretation of significance, and unsupported subgroup emphasis—raise substantive concerns for scientific accuracy, clinical interpretation, and the integrity of the digital T2D evidence base. It is respectfully requested that the authors provide clarification on these matters, and that the journal take appropriate action to issue any necessary corrections to preserve the reliability of the evidence base, ensure valid cross-study comparisons, and maintain the accuracy of the published record.

## Data Availability

No datasets were generated or analysed during the current study.
